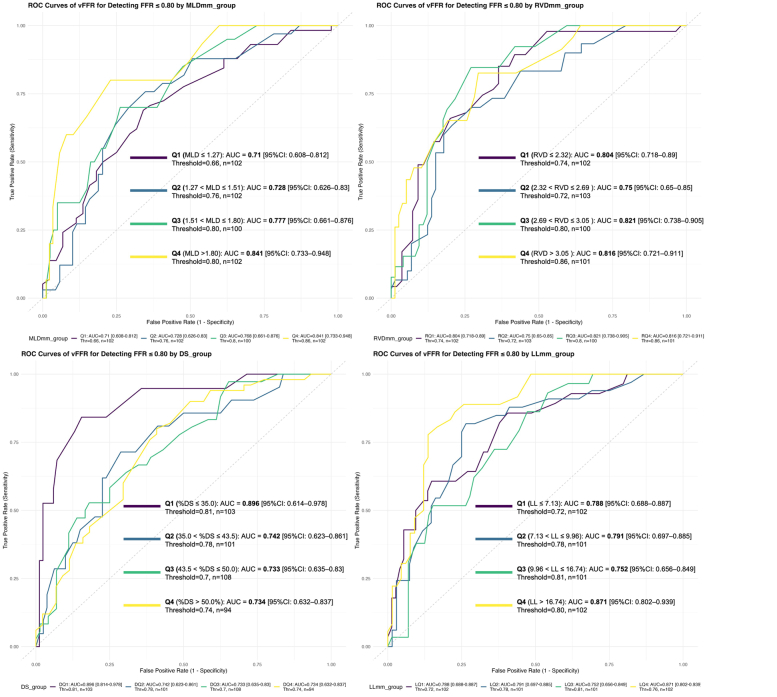# Is Minimum Lumen Diameter the Main Determinant of the Diagnostic Performance of Angiography-Based Vessel Fractional Flow Reserve?

**DOI:** 10.1016/j.jscai.2025.104055

**Published:** 2025-10-27

**Authors:** Asahi Oshima, Tsung-Ying Tsai, Chun-Chin Chang, Kai Ninomiya, Kotaro Miyashita, Akihiro Tobe, Nozomu Kanehama, Chun-Ting Shih, Adrian Bednarek, Ryo Torii, Scot Garg, Albert Chinhenzva, Yoshinobu Onuma, Patrick W. Serruys

**Affiliations:** a CORRIB Research Centre for Advanced Imaging and Core Laboratory, University of Galway, Galway, Ireland; b Department of Cardiology, School of Medicine, University of Galway, Galway, Ireland; c Cardiology Department, Galway University Hospital, Galway, Ireland; d Division of Cardiology, Cardiovascular Center, Taipei Veterans General Hospital, Taipei, Taiwan; e Cardiovascular Research Center, National Yang Ming Chiao Tung University, Taipei, Taiwan; f Institute of Clinical Medicine, National Yang Ming Chiao Tung University, Taipei, Taiwan; g Division of Cardiology, Department of Internal Medicine, Iwate Medical University, Morioka, Japan; h Department of Mechanical Engineering, University College London, London, United Kingdom; i Department of Cardiology, Royal Blackburn Hospital, Blackburn, United Kingdom; j School of Medicine, University of Lancashire, Preston, United Kingdom

**Keywords:** coronary angiography, coronary physiology, fractional flow reserve, ischemic heart disease, quantitative coronary angiography, vessel fractional flow reserve

Angiography-derived fractional flow reserve (angio-FFR) evaluates the functional significance of coronary stenoses without the need for a pressure-wire (PW) or hyperemic agents and could easily be integrated into percutaneous coronary intervention workflows to assist in lesion treatment and optimization. Its accuracy has been confirmed in observational studies, whereas the clinical utility of angio-FFR-guided percutaneous coronary intervention has been supported by randomized trials, leading to its endorsement in guidelines from the European Society of Cardiology.^1^ However, studies have reported varying diagnostic performance, with our own prospective anonymized core laboratory study showing only modest diagnostic capability of the 5 currently available angio-FFR methods and software (area under the curve [AUC], 0.73-0.79).^2^

Vessel FFR (vFFR) is one such angio-FFR method with variable diagnostic performance (AUC, 0.74-0.92). Interestingly, its ability to detect a PW-FFR ≤0.80 appears to correlate linearly with the mean vFFR of the investigated cohort (Figure 1A). Like other angio-FFR techniques, vFFR relies on solving the Gould (Q = Q_normal_⋅[1−([D_stenosis_/D_normal_ ])^4^) and Kirkeeide (ΔP = k_1_Q + k_2_Q^2^) equations using computational fluid dynamics based on angiography-derived vessel geometry; thus, it could be significantly affected by variations in vessel geometric parameters that are exponentially amplified to the second power in computational fluid dynamics formulas.^3^ Therefore, we investigated whether vessel geometries, namely, reference vessel diameter (RVD), minimal lumen diameter (MLD), percent diameter stenosis (%DS), and lesion length (LL), are associated with variations in the diagnostic performance of vFFR.

**Figure 1:**
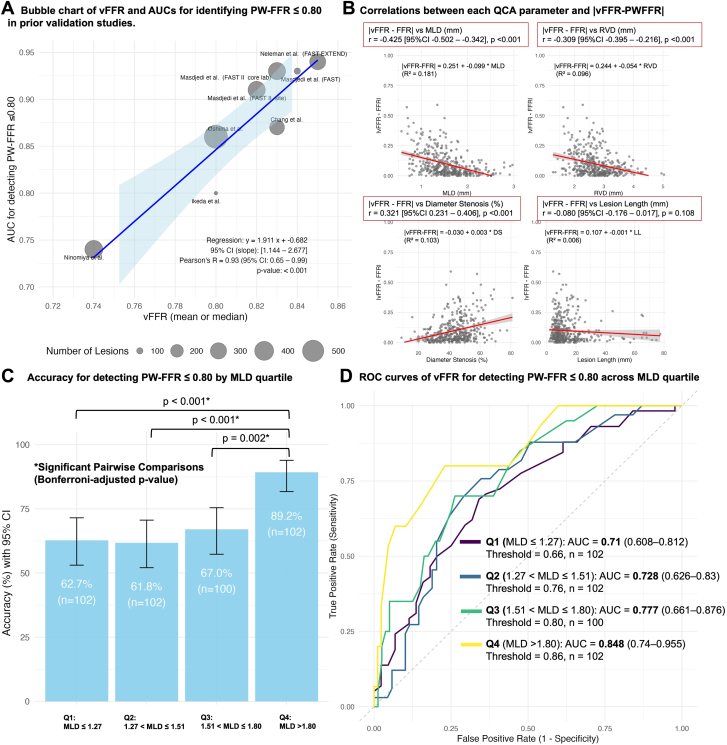
**Overall diagnostic performance and correlations of vessel fractional flow reserve (vFFR) in predictiing pressure-wire-derived FFR (PW-FFR).** (A) Bubble chart of vFFR and AUCs for identifying PW-FFR ≤ 0.80 in prior validation studies. The detailed data for the bubble charts are available in the Supplemental File S1. (B) Correlations between each QCA parameter and the absolute difference of vFFR and FFR. (C) Diagnostic performance of vFFR at a cutoff value of ≤ 0.80 for predicting PW-FFR ≤ 0.80 across MLD (mm). (D) ROC curves of vFFR for detecting PW-FFR ≤ 0.80 across MLD (mm) quartiles. AUC, area under the curve; LL, lesion length; MLD, minimum lumen diameter; NPV, negative predictive value; PPV, positive predictive value; PW-FFR, pressure-wire derived fractional flow reserve; ROC, receiver operating characteristic; RVD, reference vessel diameter; %DS, percentage diameter stenosis, vFFR, vessel fractional flow reserve.

We retrospectively analyzed a pooled population with concordant invasive physiological assessments, defined as either positive (PW-FFR ≤ 0.80 and nonhyperemic pressure ratio [NHPR] ≤ 0.89) or negative (PW-FFR > 0.80 and NHPR > 0.89) results using both hyperemic (PW-FFR) and nonhyperemic indices (instantaneous flow ratio or resting full-cycle ratio; Supplemental Figure S1). The cohort included 90 patients (103 vessels) from the retrospective Taipei Veterans General Hospital cohort and 279 (303 vessels) from the prospective multicenter ReVEAL iFR (Radiographic imaging Validation and EvALuation for Angio-iFR) trial (NCT03857503).^4^^,^^5^ Both studies complied with the Declaration of Helsinki and were approved by all institutional ethics committees. All patients provided written informed consent for participation. The patient characteristics and methodologies used for functional assessments and vFFR have been previously described.^4^^,^^5^

Angiograms were analyzed by accredited core lab analysts blinded to functional assessments using the CAAS workstation 8.5 (Pie Medical Imaging) for 2-dimensional quantitative coronary angiography (QCA) and vFFR analysis. The QCA analysis was performed using the image with the least vessel foreshortening or overlap among the 2 projections used for vFFR analysis. Correlations between the absolute difference between vFFR and PW-FFR values (|vFFR-PW-FFR|) and QCA parameters were assessed. The diagnostic accuracy of a vFFR ≤ 0.80 for detecting positive invasive physiology was evaluated across quartiles of QCA parameters. Receiver operator characteristic curve analysis was performed to identify vessels with positive invasive physiology using vFFR in each group, with the corresponding AUC values compared using DeLong test. Multiple comparisons were adjusted using Bonferroni corrections. Statistical significance was set at 2-sided *P* < .05 and performed using R version 4.4.0 (R Foundation for Statistical Computing).

A total of 406 vessels from 369 patients were included in the analysis. Of these, 234 (58%) were left anterior descending artery, 78 (19%) were circumflex artery, and 94 (23%) were right coronary artery. The mean PW-FFR, NHPR, and vFFR values were 0.83 (0.12), 0.90 (0.11), and 0.77 (0.15), respectively. Among 406 vessels, 126 (31%) had concordant positive invasive results (PW-FFR ≤ 0.80 and NHPR ≤ 0.89), and 280 (69%) had concordant negative invasive results (PW-FFR > 0.80 and NHPR > 0.89) (Supplemental Table S1; Figure 1). Of these, 106 (26%) vessels showed concordant positive results on both invasive (PW-FFR ≤ 0.80 and NHPR ≤ 0.89) and noninvasive (vFFR < 0.80) testing, 179 (44%) showed concordant negative results (PW-FFR > 0.80, NHPR > 0.89, and vFFR ≥ 0.80), and 121 (30%) showed discordance between vFFR and invasive physiological testing (Supplemental Table S1). The median RVD, MLD, %DS, and LL values were 2.69 (2.32-3.05) mm, 1.51 (1.27-1.80) mm, 43.5% (35%-50%), and 9.96 (7.13-16.74) mm, respectively.

There was a significant correlation between the QCA parameters MLD (r = −0.425, *P* < .001), followed by %DS (r = 0.321, *P* < .001) and RVD (r = −0.309, *P* < .001), and the absolute difference between vFFR and FFR (Figure 1B). When stratified by MLD quartiles, accuracy increased from 62.7% in Q1 (MLD ≤ 1.27 mm) to 89.2% in Q4 (MLD > 1.80 mm; *P* < .001). Pairwise comparisons showed significant differences between Q1 and Q4 (*P* < .001), Q2 and Q4 (*P* < .001), and Q3 and Q4 (*P* = .002, Figure 1C). There was a nonsignificant increase in AUC from 0.710 (Q1) to 0.848 (Q4) (*P* = .07, Bonferroni-adjusted *P* = .43, Figure 1D). A summary of the statistical analyses is shown in the Supplemental file.

In this study, we investigated the potential relationship between vessel geometry and the variation in diagnostic performance of vFFR. We found that MLD and %DS were significantly associated with errors in vFFR estimation, with MLD having the strongest correlation. Diagnostic accuracy improved significantly once the MLD exceeded 1.80 mm. Additionally, although statistical significance was not reached, receiver operator characteristic curve analysis showed a gradual increase in AUC values across MLD quartiles, with Q4 showing the highest diagnostic discrimination (AUC = 0.848) compared with Q1 (AUC = 0.710) (Figure 1D). Limited accuracy in contour detection for small MLDs, as shown in our previous phantom analysis, is compounded by the squared function used to calculate lumen area from MLD, which is squared again in the Lance-Gould equation, possibly explaining the amplified error.^3^ Therefore, caution is warranted when using angio-FFR in vessels with a small MLD and an intermediate range of %DS (between 35% and 50% in the current cohort), with diagnostic performance generally acceptable across different ranges of RVD and LL. Furthermore, the present study mainly included focal lesions, with a median LL of 9.96 (7.13-16.74) mm. Previous work from our group demonstrated that the physiological pattern of coronary disease, focal versus diffuse, significantly affects PW-FFR/NHPR discordance, with focal physiology more often associated with PW-FFR+/NHPR− and diffuse physiology with PW-FFR−/NHPR+.^6^ Whether vFFR maintains comparable diagnostic accuracy in long or diffuse lesions remains uncertain and warrants further investigation in future studies.

Some limitations of this study should be acknowledged. First, the sample size was relatively modest (n = 406) and may have limited the statistical power of subgroup analyses, particularly when vessels were divided into quartiles for evaluating the association between geometric parameters and vFFR diagnostic performance; the inclusion of patients from different geographic regions and the independent core lab analysis enhances the validity of our findings. Second, the diagnostic accuracy of vFFR depends on the quality of angiographic images and precise contour delineation; the angiographies included in the current study were acquired following prespecified acquisition protocols, which may increase their analyzability compared with real-world practice. In summary, the variability of vFFR performance is partly influenced by vessel geometry, with diagnostic accuracy being more reliable with larger MLDs.

## Declaration of competing interest

Patrick W. Serruys has received fees or honoraria for lectures, advisory board activities, or consulting from SMT, Novartis, and Meril Life Sciences. Scot Garg has received fees or honoraria for lectures, advisory board activities, or consulting from Biosensors International and Novartis. The other authors reported no financial interests.

## Figures and Tables

**Supplementary Figure 1:**
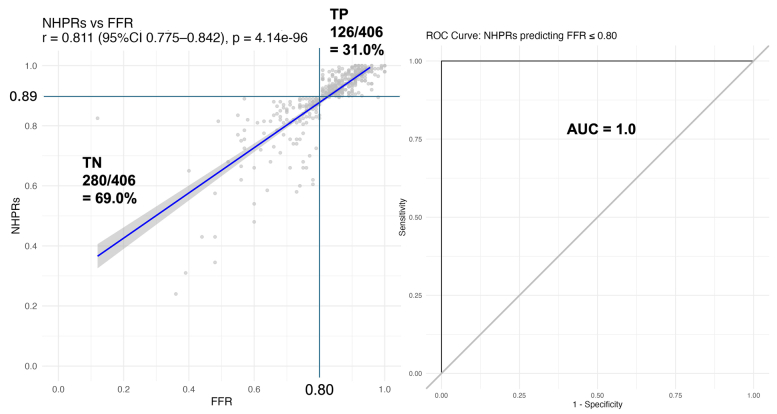


**Supplementary Figure 2:**