# Supplementary material for: Is Minimum Lumen Diameter the Main Determinant of the Diagnostic Performance of Angiography-Based Vessel Fractional Flow Reserve?
Source: J Soc Cardiovasc Angiogr Interv. 2025 Oct 27;5(1):104055. doi: 10.1016/j.jscai.2025.104055 (PMC13033794; doi:10.1016/j.jscai.2025.104055)
Supplement: Supplementary Material [file mmc1.docx]

**Supplementary file**

**Supplementary Table 1. Confusion matrix of vFFR at a cut-off value of ≤0.80 for predicting PW-FFR ≤0.80 (and NHPRs≤0.89).**

|  | **PW-FFR≤0.80**  **(and NHPRs≤0.89)** | **PW-FFR>0.80**  **(and NHPRs>0.89)** | **Total** |
| --- | --- | --- | --- |
| **vFFR≤0.80** | 106 | 101 | 207 |
| **vFFR>0.80** | 20 | 179 | 199 |
| **Total** | 126 | 280 | 406 |

NHPR: Non-Hyperaemic Pressure Ratio; PW-FFR: Pressure-Wire Derived Fractional Flow Reserve; vFFR: Vessel Fractional Flow Reserve

**Supplementary Table 2. Confusion matrix of vFFR at a cut-off value of ≤0.80 for predicting PW-FFR ≤0.80 across MLD (mm), RVD (mm), %DS (%) and LL (mm) quartile groups**

|  | Q1:  MLD ≤ 1.27 (mm)  N = 102 | Q2 :  1.27 < MLD ≤ 1.51 (mm) N = 102 | Q3:  1.51 < MLD ≤ 1.80 (mm)  N = 100 | Q4:  MLD >1.80 (mm)  N = 102 |
| --- | --- | --- | --- | --- |
| True Positive | 57 | 27 | 14 | 8 |
| False Positive | 37 | 33 | 27 | 4 |
| False Negative | 1 | 6 | 6 | 7 |
| True Negative | 7 | 36 | 53 | 83 |
|  | Q1:  RVD ≤ 2.32 mm  N = 102 | Q2 :  2.32 < RVD ≤ 2.69 mm  N = 103 | Q3:  2.69 < RVD ≤ 3.05 mm  N = 100 | Q4:  RVD > 3.05 mm   N = 101 |
| True Positive | 46 | 25 | 22 | 13 |
| False Positive | 29 | 42 | 20 | 10 |
| False Negative | 1 | 5 | 4 | 10 |
| True Negative | 26 | 31 | 54 | 68 |
|  | Q1:  %DS ≤ 35.0%  N = 103 | Q2 :  35.0% < %DS ≤ 43.5%  N = 101 | Q3:  43.5% < %DS ≤ 50.0%  N = 108 | Q4:  %DS > 50.0%  N = 94 |
| True Positive | 15 | 16 | 28 | 47 |
| False Positive | 10 | 30 | 35 | 26 |
| False Negative | 4 | 5 | 8 | 3 |
| True Negative | 74 | 50 | 37 | 18 |
|  | Q1:  LL ≤ 7.13 mm  N = 102 | Q2 :  7.13 < LL ≤ 9.96 mm  N = 101 | Q3:  9.96 < LL ≤ 16.74 mm  N = 101 | Q4:  LL > 16.74 mm  N = 102 |
| True Positive | 23 | 28 | 23 | 32 |
| False Positive | 28 | 24 | 32 | 17 |
| False Negative | 5 | 5 | 6 | 4 |
| True Negative | 46 | 44 | 40 | 49 |

%DS: Percent Diameter Stenosis; LAD: Left Anterior Descending Artery; LCX: Left Circumflex Artery; MLD: Minimum Lumen Diameter; NHPR: Non-Hyperaemic Pressure Ratio; PW-FFR: Pressure-Wire Derived Fractional Flow Reserve; RCA: Right Coronary Artery; vFFR: Vessel Fractional Flow Reserve

**Supplementary Table 3. Diagnostic performance of vFFR at a cut-off value of ≤0.80 for predicting PW-FFR ≤0.80 across MLD (mm), RVD (mm), %DS (%) and LL (mm) quartile groups**

|  | **Q1:**  **MLD ≤ 1.27 (mm) N = 102** | **Q2 :**  **1.27 < MLD ≤ 1.51 (mm) N = 102** | **Q3:**  **1.51 < MLD ≤ 1.80 (mm)  N = 100** | **Q4:**  **MLD >1.80 (mm) N = 102** | **Overall**  **p-value*** | **Significant Pairwise Comparisons**  **(Bonferroni-adjusted**  **p-value)^#^** |
| --- | --- | --- | --- | --- | --- | --- |
| **Sensitivity** | 98.3%  (90.9–99.7) | 81.8%  (65.6–91.4) | 70.0%  (48.1–85.5) | 53.3%  (30.1–75.2) | <0.001 | Q1 vs Q3 (p = 0.005)　　　　　　　Q1 vs Q4 (p <0.001) |
| **Specificity** | 15.9%  (7.9–29.4) | 52.2%  (40.6–63.5) | 66.2%  (55.4–75.7) | 95.4%  (88.8–98.2) | <0.001 | Q1 vs Q2 (p <0.001) Q1 vs Q3 (p <0.001) Q1 vs Q4 (p <0.001) Q2 vs Q4 (p <0.001) Q3 vs Q4 (p <0.001) |
| **PPV** | 60.6%  (50.5–69.9) | 45.0%  (33.1–57.5) | 34.1%  (21.6–49.5) | 66.7%  (39.1–86.2) | 0.022 | Q1 vs Q3 (p = 0.031) |
| **NPV** | 87.5%  (52.9–97.8) | 85.7%  (72.2–93.3) | 89.8%  (79.5–95.3) | 92.2%  (84.8–96.2) | 0.717 | None |
| **Accuracy** | 62.7%  (53.1–71.5) | 61.8%  (52.1–70.6) | 67.0%  (57.3–75.4) | 89.2%  (81.7–93.9) | <0.001 | Q1 vs Q4 (p <0.001) Q2 vs Q4 (p <0.001) Q3 vs Q4 (p = 0.002) |
| **Positive likelihood ratio** | 1,17  (1.02-1.33) | 1.71  (1.27-2.30) | 2.07  (1.36-3.16) | 11.6  (3.99-33.7) | - | - |
| **Negative likelihood ratio** | 0.11  (0.01-0.85) | 0.35  (0.16-0.74) | 0.45  (0.23-0.90) | 0.49  (0.28-0.84) | - | - |
|  | **Q1:**  **RVD ≤ 2.32 mm N = 102** | **Q2 :**  **2.32 < RVD ≤ 2.69 mm**  **N = 103** | **Q3:**  **2.69 < RVD ≤ 3.05 mm**  **N = 100** | **Q4:**  **RVD > 3.05 mm  N = 101** | **Overall**  **p-value*** | **Significant Pairwise Comparisons**  **(Bonferroni-adjusted**  **p-value)^#^** |
| **Sensitivity** | 97.9%  (88.9–99.6) | 83.3%  (66.4–92.7) | 84.6%  (66.5–93.8) | 56.5%  (36.8–74.4) | <0.001 | Q1 vs Q4 (p <0.001) |
| **Specificity** | 47.3%  (34.7–60.2) | 42.5%  (31.8–53.9) | 73.0%  (61.9–81.8) | 87.2%  (78.0–92.9) | <0.001 | Q1 vs Q3 (p = 0.02)  Q1 vs Q4 (p <0.001)  Q2 vs Q3 (p = 0.001)  Q2 vs Q4 (p <0.001) |
| **PPV** | 61.3%  (50.0–71.5) | 37.3%  (26.7–49.3) | 52.4%  (37.7–66.6) | 56.5%  (36.8–74.4) | 0.036 | Q1 vs Q2 (p = 0.04) |
| **NPV** | 96.3%  (81.7–99.3) | 86.1%  (71.3–93.9) | 93.1%  (83.6–97.3) | 87.2%  (78.0–92.9) | 0.378 | None |
| **Accuracy** | 70.6%  (61.1–78.6) | 76.0%  (66.8–83.3) | 76.0%  (66.8–83.3) | 80.2%  (71.4–86.8) | <0.001 | Q2 vs Q3 (p = 0.01)  Q2 vs Q4 (p = 0.001) |
| **Positive likelihood ratio** | 1.86  (1.44-2.39) | 1.49  (1.12-1.87) | 3.13  (2.08-4.71) | 4.41  (2.31-8.71) | - | - |
| **Negative likelihood ratio** | 0.05  (0.01-0.32) | 0.39  (0.17-0.91) | 0.21  (0.08-0.52) | 0.50  (0.31-0.80) | - | - |
|  | **Q1:**  **%DS ≤ 35.0% N = 103** | **Q2 :**  **35.0% < %DS ≤ 43.5%**  **N = 101** | **Q3:**  **43.5% < %DS ≤ 50.0%**  **N = 108** | **Q4:**  **%DS > 50.0% N = 94** | **Overall**  **p-value*** | **Significant Pairwise Comparisons**  **(Bonferroni-adjusted**  **p-value)^#^** |
| **Sensitivity** | 78.9%  (56.7–91.5) | 76.2%  (54.9–89.4) | 77.8%  (61.9–88.3) | 94.0%  (83.8–97.9) | 0.106 | None |
| **Specificity** | 88.1%  (79.5–93.4) | 62.5%  (51.5–72.3 | 51.4%  (40.1–62.6) | 40.9%  (27.7–55.6) | <0.001 | Q1 vs Q2 (p = 0.003)  Q1 vs Q3 (p <0.001)  Q1 vs Q4 (p <0.001) |
| **PPV** | 60.0%  (40.7–76.6) | 34.8%  (22.7–49.2) | 44.4%  (32.8–56.7) | 64.4%  (52.9–74.4) | 0.008 | Q2 vs Q4 (p = 0.014) |
| **NPV** | 94.9%  (87.5–98.0) | 90.9%  (80.4–96.1) | 82.2%  (68.7–90.7) | 85.7%  (65.4–95.0) | 0.137 | None |
| **Accuracy** | 86.4%  (78.5–91.7) | 65.3%  (55.7–73.9) | 60.2%  (50.8–68.9) | 69.1%  (59.2–77.6) | <0.001 | Q1 vs Q2 (p = 0.006)  Q1 vs Q3 (p <0.001) |
| **Positive likelihood ratio** | 6.63  (3.54–12.41) | 2.03  (1.40–2.94) | 1.60  (1.19–2.15) | 1.59  (1.23–2.05) | - | - |
| **Negative likelihood ratio** | 0.24  (0.10–0.57) | 0.38  (0.17–0.83) | 0.43  (0.23–0.83) | 0.15  (0.05–0.46) | - | - |
|  | **Q1:**  **LL ≤ 7.13 mm N = 102** | **Q2 :**  **7.13 < LL ≤ 9.96 mm**  **N = 101** | **Q3:**  **9.96 < LL ≤ 16.74 mm**  **N = 101** | **Q4:**  **LL > 16.74 mm N = 102** | **Overall**  **p-value*** | **Significant Pairwise Comparisons**  **(Bonferroni-adjusted p-value)^#^** |
| **Sensitivity** | 82.1%  (64.4–92.1) | 84.8%  (69.1–93.3) | 79.3%  (61.6–90.2) | 88.9%  (74.7–95.6) | 0.750 | None |
| **Specificity** | 62.2%  (50.8–72.4) | 64.7%  (52.8–75.0) | 55.6%  (44.1–66.5) | 74.2%  (62.6–83.3) | 0.150 | None |
| **PPV** | 45.1%  (32.3–58.6) | 53.8%  (40.5–66.7) | 41.8%  (29.7–55.0) | 65.3%  (51.3–77.1) | 0.084 | None |
| **NPV** | 90.2%  (79.0–95.7) | 89.8%  (78.2–95.6) | 87.0%  (74.3–93.9) | 92.5%  (82.1–97.0) | 0.842 | None |
| **Accuracy** | 67.6%  (58.1–75.9) | 71.3%  (61.8–79.2) | 62.4%  (52.6–71.2) | 79.4%  (70.6–86.1) | 0.06 | None |
| **Positive likelihood ratio** | 2.17  (1.55–3.05) | 2.40  (1.69–3.42) | 1.78  (1.30–2.45) | 3.45  (2.25–5.28) | - | - |
| **Negative likelihood ratio** | 0.29  (0.13–0.65) | 0.23  (0.10–0.54) | 0.37  (0.18–0.78) | 0.15  (0.06–0.38) | - | - |

**Supplementary Table 4. AUC comparison across MLD (mm), RVD (mm), %DS (mm) and LL (mm) quartile groups**

| **AUC comparison between MLD quartile groups.** | | | | | | |
| --- | --- | --- | --- | --- | --- | --- |
| Group1 | Group2 | AUC1 | AUC2 | Delta | p_raw | p_bonferroni |
| Q1 | Q2 | 0.71022727 | 0.72815108 | -0.0179238 | 0.80756131 | 1 |
| Q1 | Q3 | 0.71022727 | 0.7684375 | -0.0582102 | 0.44253425 | 1 |
| Q1 | Q4 | 0.71022727 | 0.84750958 | -0.1372823 | 0.07123011 | 0.42738067 |
| Q2 | Q3 | 0.72815108 | 0.7684375 | -0.0402864 | 0.59532106 | 1 |
| Q2 | Q4 | 0.72815108 | 0.84750958 | -0.1193585 | 0.11677323 | 0.70063936 |
| Q3 | Q4 | 0.7684375 | 0.84750958 | -0.0790721 | 0.31112081 | 1 |
| **AUC comparison between RVD quartile groups.** | | | | | | |
| Group1 | Group2 | AUC1 | AUC2 | Delta | p_raw | p_bonferroni |
| RQ1 | RQ2 | 0.80367505 | 0.74977169 | 0.05390336 | 0.424349 | 1 |
| RQ1 | RQ3 | 0.80367505 | 0.8214657 | -0.0177906 | 0.77138527 | 1 |
| RQ1 | RQ4 | 0.80367505 | 0.82441472 | -0.0207397 | 0.75151393 | 1 |
| RQ2 | RQ3 | 0.74977169 | 0.8214657 | -0.071694 | 0.28149588 | 1 |
| RQ2 | RQ4 | 0.74977169 | 0.82441472 | -0.074643 | 0.28982857 | 1 |
| RQ3 | RQ4 | 0.8214657 | 0.82441472 | -0.002949 | 0.96354123 | 1 |
| **AUC comparison between %DS quartile groups.** | | | | | | |
| Group1 | Group2 | AUC1 | AUC2 | Delta | p_raw | p_bonferroni |
| DQ1 | DQ2 | 0.90601504 | 0.7422619 | 0.16375313 | 0.02653306 | 0.15919839 |
| DQ1 | DQ3 | 0.90601504 | 0.73263889 | 0.17337615 | 0.00775469 | 0.04652814 |
| DQ1 | DQ4 | 0.90601504 | 0.73431818 | 0.17169686 | 0.01071113 | 0.06426681 |
| DQ2 | DQ3 | 0.7422619 | 0.73263889 | 0.00962302 | 0.90240029 | 1 |
| DQ2 | DQ4 | 0.7422619 | 0.73431818 | 0.00794372 | 0.92112062 | 1 |
| DQ3 | DQ4 | 0.73263889 | 0.73431818 | -0.0016793 | 0.98147403 | 1 |
| **AUC comparison between LL quartile groups.** | | | | | | |
| Group1 | Group2 | AUC1 | AUC2 | Delta | p_raw | p_bonferroni |
| LQ1 | LQ2 | 0.78764479 | 0.80392157 | -0.0162768 | 0.8145384 | 1 |
| LQ1 | LQ3 | 0.78764479 | 0.75239464 | 0.03525015 | 0.61951529 | 1 |
| LQ1 | LQ4 | 0.78764479 | 0.87058081 | -0.082936 | 0.18085708 | 1 |
| LQ2 | LQ3 | 0.80392157 | 0.75239464 | 0.05152693 | 0.45078857 | 1 |
| LQ2 | LQ4 | 0.80392157 | 0.87058081 | -0.0666592 | 0.25705623 | 1 |
| LQ3 | LQ4 | 0.75239464 | 0.87058081 | -0.1181862 | 0.05229996 | 0.31379977 |

DeLong’s test was used to compare AUC values between quartile groups. Multiple comparisons were corrected using Bonferroni methods. AUC: Area Under the Curve; CI: Confidence Interval; %DS: Percentage Diameter Stenosis; LL: Lesion Length; MLD: Minimum Lumen Diameter; NPV: Negative Predictive Value; PPV: Positive Predictive Value; PW-FFR: Pressure-Wire Derived Fractional Flow Reserve; ROC: Receiver Operating Characteristic; RVD: Reference Vessel Diameter; vFFR: Vessel Fractional Flow Reserve

**Supplementary Table 5. Summary of AUCs for detecting PW-FFR ≦0.80 using vFFR.**

| Study | N (lesions) | Characteristics of patients | PW-FFR value  Mean (±) or Median [Q1-Q3] | vFFR value  Mean (±) or Median [Q1-Q3] | AUC for detecting  PW-FFR≦0.80 | Reference |
| --- | --- | --- | --- | --- | --- | --- |
| Masdjedi et al. (FAST) | 100 | stable angina or NSTEMI | 0.82±0.08 | 0.84 ± 0.07 | 0.93 (95%CI: 0.88-0.90) | EuroIntervention  . 2020 Sep 18;16(7):591-599. doi: 10.4244/EIJ-D-19-00466. |
| Neleman et al.  (FAST EXTEND) | 294 | stable angina, unstable angina, and NSTEMI | 0.84 [0.79-0.90] | 0.85 [0.80-0.90] | 0.94 (95%CI: 0.92-0.97) | JACC Cardiovasc Imaging  . 2021 Feb;14(2):504-506. doi10.1016/j.jcmg.2020.08.006. |
| Chang et al. | 258 | chronic coronary syndrome, unstable angina or NSTEMI | 0.84 ± 0.08 | 0.83 ± 0.10 | 0.87 (95%CI: 0.83–0.92) | Front Cardiovasc Med  . 2021 Jun 30:8:707454. doi: 10.3389/fcvm.2021.707454. |
| Masdjedi et al.  (FAST II) core lab | 334 | chronic coronary syndromes, unstable angina or NSTEMI | 0.83±0.08;  0.84 [0.78-0.89] | 0.83±0.09; 0.85 [0.78-0.89] | 0.93 (95%CI: 0.90-0.96) | EuroIntervention  . 2022 Apr 22;17(18):1498-1505. doi: 10.4244/EIJ-D-21-00471. |
| Masdjedi et al.  (FAST II) site | 334 | chronic coronary syndromes, unstable angina or NSTEMI | 0.83±0.08;  0.84 [0.78-0.89] | 0.82±0.10;  0.84 [0.79-0.89] | 0.91 (95%CI: 0.88-0.94) | EuroIntervention  . 2022 Apr 22;17(18):1498-1505. doi: 10.4244/EIJ-D-21-00471. |
| Ikeda et al. | 90 | stable angina or NSTEMI | 0.78 [0.72-0.84] | 0.80 [0.66–0.87] | 0.80 (95%CI: 0.70-0.90) | Catheter Cardiovasc Interv  . 2023 Jan;101(1):72-78. doi: 10.1002/ccd.30511. |
| Ninomiya et al. | 359 | chronic coronary syndrome | 0.82 ± 0.12 | 0.74± 0.15; 0.76 [0.64-0.85] | 0.74 (95%CI: 0.68-0.79) | JACC Cardiovasc Interv. 2023 Jul 24;16(14):1778-1790. doi: 10.1016/j.jcin.2023.04.026. |
| Oshima et al. | 586 | chronic coronary syndromes, unstable angina or NSTEMI | 0.81 ± 0.09;  0.82 [0.75-0.88] | 0.80 ± 0.10;  0.82 [0.74-0.87] | 259 (44%) | Catheter Cardiovasc Interv. 2025 Jul;106(1):207-217. doi: 10.1002/ccd.31534. |

AUC: area under the curve; NSTEMI: non-ST-elevation myocardial infraction; PW-FFR: pressure wire fractional flow reserve; vFFR: vessel fractional flow reserve.

**Supplementary Figure 1.**

**Constructed Optimal Benchmark of Hyperaemic (PW-FFR) and Non-Hyperaemic (NHPR) Pressure-Derived Physiological Assessment
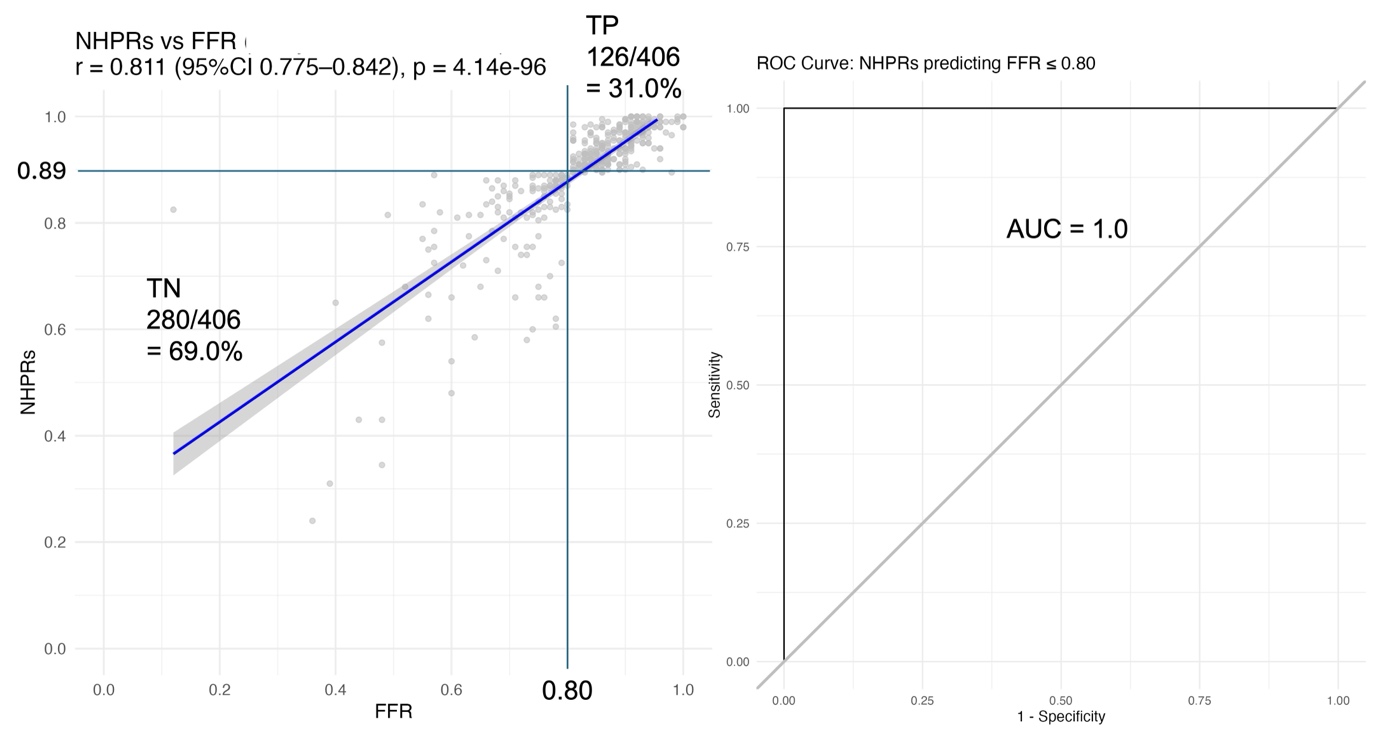
**

**Left: The scatterplot of the relationship between NHPRs and PW-FFR in concordant cases. Right: A ROC curve shows a theoretical AUC of one.**

**Supplementary Figure 2. ROC curves of vFFR for detecting PW-FFR ≤ 0.80 across MLD (mm), RVD (mm), %DS (mm) and LL (mm) quartile groups**

**
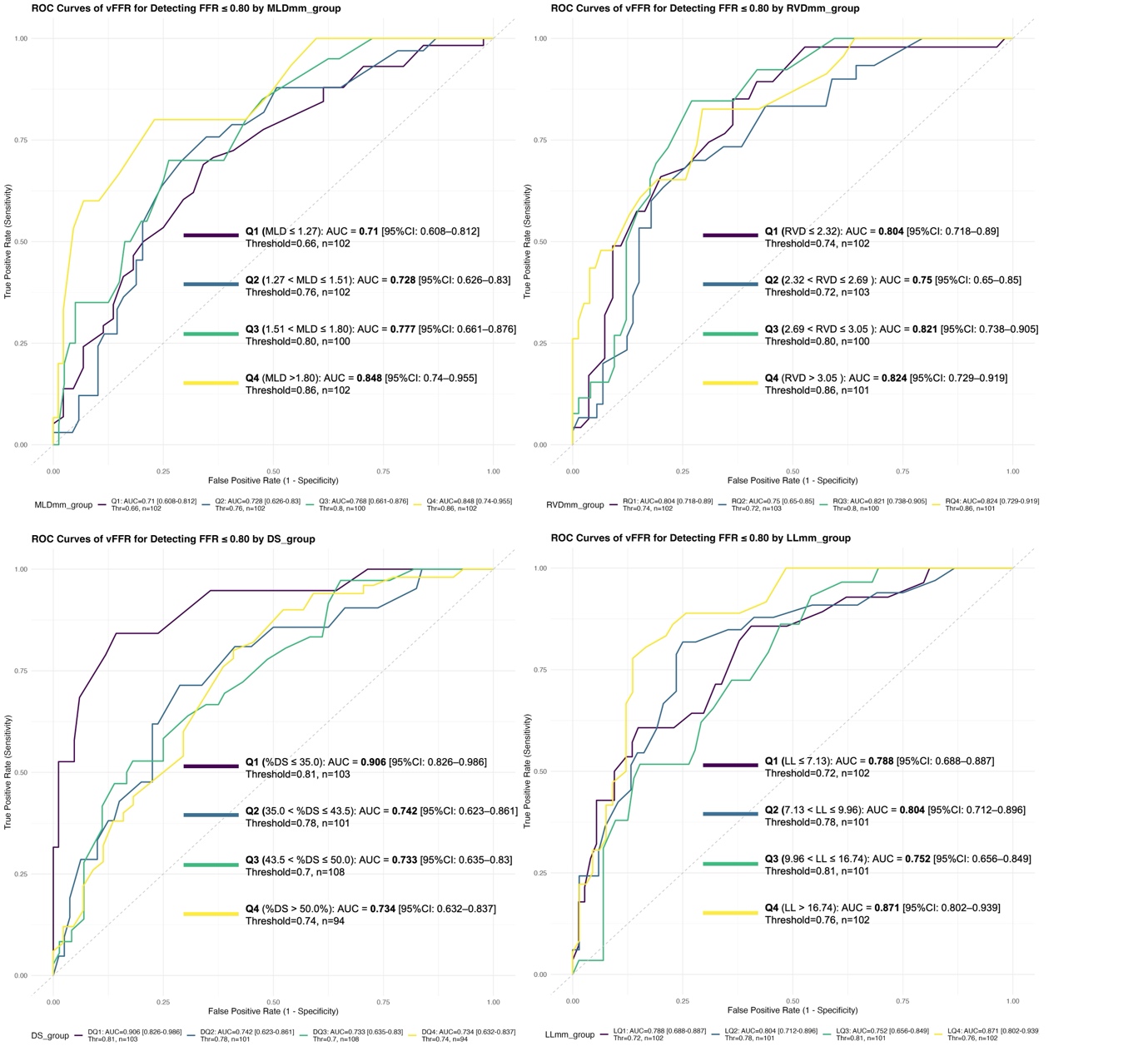
**

AUC: Area Under the Curve, CI: Confidence Interval, LL: Lesion Length, MLD: Minimum Lumen Diameter, NPV: Negative Predictive Value, PPV: Positive Predictive Value, PW-FFR: Pressure-Wire Derived Fractional Flow Reserve, ROC: Receiver Operating Characteristic, RVD: Reference Vessel Diameter, %DS: Percentage Diameter Stenosis, vFFR: Vessel Fractional Flow Reserve
